# Health promotion roles shaped by professional identity: an ethnographic study in the Netherlands

**DOI:** 10.1093/heapro/daad195

**Published:** 2024-01-13

**Authors:** F van Heteren, N J Raaphorst, J M Bussemaker

**Affiliations:** Department of Public Health and Primary Care/Health Campus, Leiden University Medical Centre, Turfmarkt 99, 2511 DP The Hague, The Netherlands; Faculty of Governance and Global Affairs, Institute of Public Administration, Leiden University, 2511 DP The Hague, The Netherlands; Faculty of Governance and Global Affairs, Institute of Public Administration, Leiden University, 2511 DP The Hague, The Netherlands; Department of Public Health and Primary Care/Health Campus, Leiden University Medical Centre, Turfmarkt 99, 2511 DP The Hague, The Netherlands; Faculty of Governance and Global Affairs, Institute of Public Administration, Leiden University, 2511 DP The Hague, The Netherlands

**Keywords:** professional identity, health promotion roles, psychosocial care, frontline professionals, qualitative research

## Abstract

How frontline care professionals interpret and fulfill their health promotion roles is of great importance for the health of the vulnerable clients they work with. While the literature on health promotion is limited to describing the roles of healthcare professionals, this study examines the health promotion roles held by various frontline professionals when working with clients with combined psychosocial problems and how this is associated with professional identity. Based on ethnographic data from Dutch frontline professionals in social welfare, general healthcare and mental healthcare, this article shows how various frontline professionals promote health by reframing and customizing health problems and that this is associated with how they identify as pragmatic or holistic professionals.

**Contribution to Health Promotion**

- Frontline professionals behave according to two health promotion roles: reframing and customized health promotion.
- Health promotion roles differ based on how professionals manage complexity and client autonomy, and how they involve the client context.
- Health promotion roles relate to how professionals identify as pragmatic or holistic professionals.

## INTRODUCTION

Given their position at the frontline of care, how professionals in social welfare, general and mental healthcare fulfill their responsibilities is of direct importance to the clients they work with (Zacka, 2017; Von Greiff *et al*., 2020). The health of the population is influenced by social determinants throughout the life course. This implies that efforts aimed at promoting health has in recent decades become a collective responsibility, needing a comprehensive approach involving various sectors and diverse partners, rather than being solely the purview of the (mental) healthcare sector (Shields-Zeeman, 2021). It is therefore important to look not only at the health promotion roles, including prevention and changing behaviors of individuals with respect to their health, of medical professionals such as general practitioners (GPs) and practice nurses, but also at the roles of other types of professionals (McAvoy, 2000; Geense *et al.,* 2013; Kemppainen *et al.,* 2013). Insight into the different health promotion roles of professionals is relevant because this may help them to work together to promote the health of vulnerable clients suffering from combined problems.

A professional role embodies the perceived professional tasks and functions that are specific to a professional group. The role that professionals play, or what professionals value and how they behave, is shaped by the ways in which they are socialized in different professional contexts (Weis and Schank, 2002; Møller, 2021). We are aware of the literature on professional logics (Abbott, 2014; Cecchini and Harrits, 2022), but in this article, we adopt the approach of examining professional identity. Professional socialization is a process of learning, interacting, developing and adapting (Dinmohammadi *et al.,* 2013) and contributes to the formation of identity. Identity is someone’s self-definition, which answers the question ‘*Who am I?*’ or ‘*Who are we*?’ (Ashforth and Schinoff, 2016). In turn, professional identity is defined as ‘the attributes, values, knowledge, beliefs and skills shared with others within a professional group’ (Adams *et al*., 2006). As such, we expect that professional identity, or subjective self-conceptualization associated with the work role (Adams *et al.,* 2006), shapes professionals’ health promotion roles (Agresta, 2004).

Our research question is therefore as follows: What kind of health promotion roles do professionals in healthcare, mental healthcare and social welfare have and how are these shaped by their professional identity? This question will be answered through an ethnographic study of various frontline professionals in social welfare, mental healthcare and general healthcare who work in health promotion with clients who have psychosocial problems.

This study contributes to the professional health promotion literature in two ways. First, earlier health promotion studies focus on medical professionals such as GPs and nurses (a.o. Geense *et al.,* 2013). Our research includes various types of frontline professionals, in mental healthcare and social welfare, but also in general healthcare, all of whom are involved with clients with psychosocial problems. Second, while empirical studies on health promotion have offered descriptions of different professional health promotion roles (e.g. Geense *et al.,* 2013), these are only described as tasks. In this research, we offer a conceptualization of health promotion roles and how these are shaped by professional identity. As professional roles develop within specific professional contexts and workplaces with distinct sets of values, knowledge and skills (Barnhoorn *et al.,* 2022), we examined the professional embeddedness of health promotion roles. Understanding how health promotion roles are shaped by professional identity is furthermore relevant for discussions among professionals, managers and policymakers on how to ensure quality and continuity in care. To this end, we studied professionals over a longer period of time to examine how and why professionals take on health promotion roles.

In the following sections we will present our theoretical framework delineating the core concepts of this study, after which we will describe our ethnographic methodology and research context. We will then present our findings and conclude with theoretical and practical implications.

## THEORETICAL FRAMEWORK

### Professional health promotion roles

Health promotion is a community-based and collaborative practice based on social and health policies (Baisch, 2009), which includes prevention and behavioral change (Kemppainen *et al.,* 2013) with the aim of giving people control over their own lives (Labonte, 1994). It is also seen as preventing or minimizing risks or risky behavior (Cecchini, 2021) pertaining to the health risks of clients construed as high risk, while professionals working in health and social welfare are seen as ‘risk-minimizing agents’ (Cecchini, 2018). In line with this, professional health promotion is regarded as promoting clients’ action competences to minimize risk (ibid.). A professional role is understood as the perceived professional tasks and functions that are characteristic of a professional group (Agresta, 2004). In our study, health promotion roles are understood as how professionals perform their role in relation to the client through different activities, responsibilities and tasks that are performed aimed at improving the health of clients (Tannahill, 1985; Geense *et al*., 2013).

Professional health promotion roles have been studied for medical professionals such as GPs (McAvoy *et al*., 1999; Geense *et al*., 2013) and nurses (see McAvoy *et al.*, 1999; McKinlay *et al.*, 2005; Geense *et al*., 2013; Kemppainen *et al.*, 2013), with studies exploring professionals’ perceived approaches and attitudes towards health promotion without observing their actual roles. Their health promotion tasks (that together make up a role) range from making an enquiry about a client’s lifestyle, prevention, providing information, advising, referring and actively screening for a disease (McAvoy *et al*., 1999; McKinlay *et al.*, 2005; Geense *et al*., 2013) to modifying behaviors (Brotons *et al*., 2005). Existing literature on professional health promotion roles is highly descriptive in character and lacks analytical precision. Based on an analysis of this literature, we found that health promotion roles differ on three dimensions related to the type of involvement, perceived abilities and perceived importance in health promotion.

#### Type of involvement

The literature shows that health promotion consists of several types of involvement, namely, working *reactively* and working *proactively*. Reactive involvement means that professionals respond to clients’ clearly expressed or specific symptom or problem (McAvoy, 2000; Kemppainen *et al.,* 2013). As such, during illness visits or conversations regarding specific symptoms, professionals educate or advise their clients about behavior, lifestyle or possible risks (McAvoy *et al*., 1999). Reactive health promotion among nurses is focused on risk-specific practices related to disease in favor of behavioral, disease-focused, lifestyle-oriented determinants of health. Such strategies may fail to incorporate broader societal dimensions of health promotion (Runciman *et al.*, 2006; Whitehead, 2006; Casey, 2007). *Proactive* involvement is concerned with assessing risks and counseling, for example, during routine checkups (McAvoy *et al.*, 1999; Kemppainen *et al.*, 2013). In proactive involvement, professionals aim to work as teachers to educate their patients (McAvoy *et al.*, 1999). The main difference with reactive is that in proactive involvement, it is not necessary for clients to have any worrying symptoms to react on.

#### Perceived abilities

Perceived *abilities* in health promotion hold that professionals relate to their abilities and responsibilities in health promotion. On the one hand, professionals feel that health promotion is part of their job and they are *able*, *skilled* or *responsible* to help clients to stay or become healthy. Professional knowledge about health promotion activities helps to make them feel that they are able and/or responsible. On the other hand, professionals may feel that they are *unable*, unskilled and/or irresponsible for health promotion (Geense *et al.*, 2013). As Geense and colleagues (2013) mention, unable professionals could ignore health promotion activities, because it is up to other stakeholders to take responsibility. Moreover, some professionals only feel able to promote health when they can do so in collaboration with other stakeholders. For example, by confirming or supporting the plans made between the client and a colleague (ibid.), or by empowering individuals or communities (Kemppainen *et al.*, 2013).

#### Perceived importance

Perceived *importance* in health promotion means that professionals relate to whether health promotion is a worthwhile part of their job. Professionals may *emphasize* health promotion as a key component of their work (McAvoy, 2000). They are motivated or willing to promote health, which can be shown by offering support to other professionals working on health promotion. Professionals may also be *skeptical* about health promotion and its results and effects (Geense *et al.,* 2013), because they expect it will not make a difference. For example, while they try to motivate clients as much as they can, they may, due to low expectations, not refer them to other professionals that could further help (ibid.).

### Professional identity

Drawing on identity theory, the individual’s sense of self consists of a personal identity with characteristic attributes such as gender or age and a social identity including categories of people that may include nationality or a team member. A person is therefore a unique individual and socially they are part of a group (Ashforth, 2000). Individuals give meaning to their identity through interaction with others (Weick, 1995). Identity is thus a relational concept and is formed, among other things, through comparison (Ashforth *et al*., 2008). Identity construction is the process through which actors come to define who they are, and identification is the extent to which one internalizes an identity as a—partial—self-definition (Ashforth and Schinoff, 2016). Professional identities are often robust due to clear standards (Ashforth and Schinoff, 2016). For example, a professional category such as doctor becomes meaningful in relation to other professionals in the category of social worker or bureaucrat (Ashforth, 2000). Such well-defined groups tend to be exclusive, concrete and context-specific with clear goals, norms, member interdependencies and interactions between them (ibid., Ashforth, 2000; Adam *et al*., 2006; Pratt *et al*., 2006). Professional identity is constructed in interaction with others as it relates to ‘how people compare and differentiate themselves from other professional groups’ (Adams *et al*., 2006). Professional groups may possess various professional identities (Brown and Humphreys, 2006; McDonald *et al.*, 2008). Based on this literature we expect that professional identities often align with professional groups, but how they do so must be empirically studied.

In this study, a professional identity refers to an individual’s self-definition as a member of a profession (Ibarra, 1999; Adams *et al*., 2006; Chreim *et al.*, 2007), and it includes how one interprets one’s professional goals, values, beliefs, norms and interaction styles (Burke and Stets, 2009). Every action, speech or thought we engage in can be a manifestation of how we define ourselves as professionals (Alvesson *et al*. 2008), and how we behave in practice with others can give rise to professional identity (Weick 1996; Chreim *et al*., 2007; Touati *et al*., 2019). Moreover, individuals assign different meanings to their identity, and professionals can have multiple identities (Ashforth *et al.,* 2008; Ashforth and Schinoff, 2016), depending on the context and their multiple professional roles (Mak *et al.,* 2022). We acknowledge that connections exist between professional identities and other identities, though we do not discuss them in this paper.

Literature on identity of professionals involved in health promotion shows distinct professional identities for various types of professionals. GPs are said to include patient-centeredness, conceiving of the patient as a whole person and being an active participant in a relationship of equals, which might encompass delivering personalized healthcare (McDonald *et al*., 2008). Compared to GPs’ strong professional identities, social welfare professionals’ identities have been exposed to ambivalence towards recognition of their occupations enabled by New Public Management (NPM) and gendered presumptions emerging as factors in undermining the stability of professions in social welfare (Healy, 2009). Especially in interprofessional settings, their professional identity is unstable, thereby challenging their health promotion contributions (Bark *et al*., 2023). Professionals in mental healthcare prefer dialogical approaches for embracing their sameness with clients, which is seen both as an opportunity to connect deeply as well as a risk of exposing the limitations of professional expertise in health promotion (Schubert *et al*., 2021).

Based on identity literature, we expect that professional identity is central to how professionals interpret and play their roles in health promotion (i.e. Weick, 1996; Weis and Schank, 2002; Chreim *et al.*, 2007; Touati *et al.*, 2019; Møller, 2021). The professionals in our sample comprise a heterogenous social group when looking at their educational background, work tasks, terms of employment and income (Bourdieu, 1984; Ilsvard and Møller, 2015). Theoretically, we therefore assume that professionals with different professional backgrounds identify differently and consequently promote health differently.

## METHODOLOGY

### Research setting

The study was conducted with frontline professionals in social welfare, and general and mental healthcare in the Dutch city of The Hague with a population of approximately 500 000 people. In this city, psychosocial problems are disproportionally common, particularly among low-income residents (Parbhudayal, 2021). A Dutch Health Policy Document addresses health issues from a comprehensive standpoint, transcending domains, embracing the ‘Health in All Policies’ approach. Both the national and local governments collaborate over an extended period, prioritizing prevention and well-being (Shields-Zeeman, 2021). In line with these developments, the Hague municipal health service and he municipality work with local partners towards ambitions in health promotion concerning stimulants and health and mental resilience and psychological health (Actieprogramma Preventie, 2020; Rijksinstituut voor Volksgezondheid en Milieu, 2020). In this action program, several types of frontline professionals are asked to work with many stakeholders to care for the population. The program suggest that a local approach is useful and that frontline professionals have broad health views and knowledge, and that they exhibit creativity and entrepreneurship (Actieprogramma Preventie, 2020). Hence, this setting in which much is asked of several types of frontline professionals concerning health promotion is well suited for empirically studying professional health promotion roles.

### Research strategy and data collection

This research is ethnographic, which means that it is a study about a group of people and their lifestyle studied in their natural environment (Ybema and Kamsteeg, 2009; Cecchini, 2018). The goal of this study is to uncover how frontline professionals perceive and act on health promotion roles and professional identity. With an ethnographic strategy we can observe these professionals’ behavior (Geertz, 1974; Cecchini, 2018), which is necessary to gain insight into how professionals give substance to their role.

This study uses abductive logic, which combines deductive and inductive reasoning in an iterative process. It moves back and forth between theory and empirical observations (Schwartz-Shea and Yanow, 2013; Meyer and Ward, 2014). In this study, abduction starts with an empirical question about professional identity theory and leads to expectations about health promotion roles. Moreover, abduction involves describing and understanding the world from the respondents’ perspective and then deriving a scientific explanation (Meyer and Ward, 2014).

Respondents are frontline professionals in social welfare, and mental and general healthcare working with clients with psychosocial problems in The Hague. Theoretically, this selection is relevant to study because these professionals are said to work together in an interprofessional setting and their health promotion roles may complement, conflict or overlap. These professionals were chosen, because they are all encouraged to engage in health promotion in their work through the ‘health in all policies’ approach (Shields-Zeeman, 2021). All respondents handle many cases per day, resulting in a considerable number of observed interactions. All fieldwork was conducted by the lead author. This study aimed to gather in-depth and context specific insight into care professionals. We used a sample consisting of six main respondents working in three organizations: two GPs, two professionals in social welfare and two mental healthcare professionals to ensure valuable insights into the complexities and nuances of professional identities and professional roles in health promotion in their specific organizational settings (Møller, 2018). We studied them intensively in interaction with their colleagues and stakeholders. All professionals have been performing frontline work for many years (see Supplementary Appendix A). We studied frontline professionals at work with clients where the professional identified or suspected combined problems (i.e. problems for which professional help traverses professional fields). Our aim was not to generalize to a wider population of frontline professionals, but to gain an in-depth understanding of how professional identity shapes their health promotion roles.

The lead author gained access to these professionals by using their network built up during earlier research. Potential respondents or their supervisors were emailed requesting discussing participation in this research. The professionals were eager to learn from reflecting on their work and welcomed us openly.

The lead author conducted ethnographic fieldwork including participant observation, informal conversations and semi-structured interviews to gain insight into respondents’ health promotion roles and professional identities. Fieldwork allowed the researcher to be present at the professionals’ work location and observe the phenomena of interest where they unfold (Spradley, 2016). While perceptions about health promotion roles have been grasped by interviews before (Geense *et al.,* 2013), how roles are practiced can be tacit and hard to articulate. Roles are therefore best observed over a longer period of time in their natural setting (Walshe *et al.*, 2012; Zahle, 2012). The lead author conducted two rounds of observations during which the operationalized health promotion roles (see Section 2.1 and Supplementary Appendix B) as well as our definitions of health promotion roles and professional identity were used as sensitizing concepts.

During observations, interest was taken in how professionals behave in health-promoting consultations with clients and how they reflect on their behavior. During informal conversations and interviews, follow-up questions were asked that give insight into how professionals identify, what they value and how they interpret their interaction styles. During the fieldwork, the researcher was able to talk with the main respondents’ colleagues and others involved in the care process. Before starting each round of data generation, the observation guide and conversation scheme based on sensitizing concepts were updated (see Appendices B and D) to plan and steer the process, while still allowing for inductive findings and adjustments. The observations and interviews were conducted between January 2022 and January 2023 at the work location of the respondents (i.e. consulting rooms, house visits, team rooms). Field notes were taken during the observations and were written out in detailed reflections after or in between observations (i.e. when professionals did administrative work). Later, field notes were re-written digitally by filling in the gaps of the first descriptions (Spradley, 2016). Semi-structured interviews were audiotape-recorded and transcribed verbatim. All data were imported into ATLAS.ti version 9 for further analysis. All respondents gave informed consent. This study was registered and approved by the medical Ethics Committee of [anonymized].

The role of participant observer was taken with varying degrees of participation. This means that sometimes the researcher would be more participative, for example, by joining in the conversation during lunch. At other times, the context would allow for a more distant position, such as during a client consultation. This means that the researcher had to be flexible and act in accordance with signals given by the respondents. The researcher is thereby an instrument of data generation and data analysis (Bernard, 2017). The researcher spent a considerable amount of time in the field (34 days or 150 h, Supplementary Appendix C) with the goal of decreasing the reactivity of those observed to the presence of the researcher (ibid.), and the researcher wrote reflections about their positioning in the field.

### Analysis

The lead author first developed stories for each respondent based on the health promotion roles observed during fieldwork. A story consists of excerpts of field notes taken on health promotion during one consultation between a client and a professional, sometimes these are accompanied by respondents’ reflections.

After open coding and discussions with the second author, the lead author further specified the initial themes that were close to the empirical data. During more focused coding, the first author explicitly looked for health promotion roles and professional identities. During the abductive thematic analysis (Braun and Clarke, 2006) the authors confronted the initial findings with the definitions of health promotion roles, and professional identity and used them as sensitizing concepts. The authors then built a typology. By taking an overarching view, constant comparison (Schwartz-Shea and Yanow, 2013) between and within professional groups has been central in our analysis. The lead author used conversations with the main respondents and their colleagues to member check and search for alternative explanations. The respondents reacted to what the author observed and triangulation took place by comparing observational and conversational data (Schwartz-Shea and Yanow).

The final result shows two types of health promotion roles and two types of professional identities. The authors disentangled professional identities and professional roles in order to study the mechanisms between them. Our coding table (Supplementary Appendix E, on persona) gives insight into how respondents from various professional groups differ in their professional identities and how they fulfill their health promotion roles.

The next section outlines the main types of professional identity and professional roles in health promotion. Examples are based on the empirical data, with references made to the theoretical framework in italics.

### Health promotion roles

#### Role 1: reframing health promotion

In general, in the health promotion role of *reframing a client’s care needs* professionals are observed to reframe clients’ needs into something specific which they can work with by setting boundaries. Even though the respondent may observe the complexity of the problem, they demarcate the problem into a specific aspect which is prioritized. Ultimately, health promotion may be focused on problems for which clients did not plan an appointment or ask for help with, and it remains unclear whether clients see the prioritized aspect as a problem. In this role we see that when professionals do not necessarily react to the worries as presented by the client, they are mostly involved *proactively*. However, these professionals work *reactively* with problems that they observe themselves. Moreover, when reframing, professionals have in common that they find it *important* to act in a health-promoting way and that they experience the *ability* to promote health when a problem is close to their professional expertise. When professionals are aware of the broader problems presented by the client, they may not go into aspects of these problems right away and/ or they may refer clients to another professional, as is shown in the empirical examples below.

**‘Then back to contraception, it would be very problematic for you to get pregnant again’. (**respondent A, GP)

Before the client comes in, the respondent explains that the client did not want to tell the assistant why they are here today. However, the respondent knows that the client had an unwanted pregnancy and an abortion a while ago. Respondent: ‘*What can I do for you?*’ Client: ‘*I’m angry and lonely and it cannot go on like this. It is very private, but I came here anyway. I’m so tired and I cannot sleep. Do you see these bags under my eyes? I have a problem*.’ Respondent: ‘*Do you want to tell us about your problem?*’ Client: ‘*Yes, I’m renting this house together with a friend (…). But she left and I have to pay our rent. Now sometimes I do not have enough money to feed my child. I do not know what to do. My kids suffer from it, they are not doing well at school. (…) It hurts that I cannot take good care of them*.’ Respondent: ‘*I understand that you don’t feel good and that is not a good situation for your children. (…) Stress all day, they also feel that. And I heard you had an abortion recently? Do you now use contraception?*’ Client: ‘*Yes, but no….*’ Respondent: ‘*We could talk more about this later. How can I help you now?*’ The client stresses that her financial situation is very important for her, as is the urgency of cheaper housing. The respondent explains that a colleague, a practice nurse, has more time to help her with that. ‘*She knows about different money streams, because you know that medicines will not help in this case.*’ The client nods understanding. ‘*Then back to contraception, it would be very problematic for you to get pregnant again*.’ The respondent proposes a coil as the best option and asks if the client wants to think about it at home. After the consultation, the respondent told me that the client is ‘*at high risk of unwanted pregnancy*’, which could increase her problems.

In this example, respondent A reframes social and financial problems as expressed by the client into a lifestyle or contraception problem. Therefore, this respondent decides to *proactively* take the lead in the conversation by focusing on contraception. Contraception is something medical which he is *able* to fix and it is close to his professional expertise. Fixing this is *important* because it could, in the respondent’s opinion, prevent more problems from developing.

Professionals performing a reframing health promotion role seem to understand the complexity and broadness of the problems presented by clients, but they do not feel able or motivated to help with problems beyond their expertise.

#### Role 2: customized health promotion

Customized health promotion happens in close relationship with the client and their environment. In this role, the client is given the opportunity to take the lead and the responsibility in expressing the problems. As such, we see that in this role professionals tend to work *reactively* since they react to the very problems that are expressed by clients. However, professionals could still be *proactive*, when they propose solutions and try to educate patients. A central aspect of customized health promotion is that professionals ask in-depth follow-up questions because they find it *important* to listen and to avoid steering away from what clients express as important. Professionals are *able* to be responsive by crossing professional boundaries. In short, client autonomy is central and professionals follow the clients’ needs and solutions (respondent E, social worker).

**‘*I work differently with every client*’** (respondent D, mental health worker)

Respondent D brought an SOS tracker for the client, who seems pleased with it. With this, the client can call the mental healthcare emergency line when needed. The professional and client test the device together. A burden seems to fall off the client. Respondent to client’s partner: ‘*Do you want to have a look?*’ Last week the partner messaged that the client was not doing well. ‘*He heard voices and saw delusions, but now he is a bit calmer. When she [client’s partner] messages, then something is really wrong*.’ By using a color system, the professional discusses with the client how he feels and how they can try to prevent him from feeling that bad again. Before we leave, the respondent asks the partner how she thinks he’s doing. After the house visit, the respondent explains that they work very differently with every client. ‘*With this man I was childish, which is what he needs for his fears.’*

The above example of customized health promotion is exemplary of respondents who find it *important* to *react* to client preferences by listening closely, by collaborating with other involved professionals and in teamwork with the client environment. Professionals are *able* to take the care question seriously by taking time to figure out together with the client environment what else may play a role and how to prevent future crises.

#### Additional health promotion role dimensions

Our analysis provides reason to distinguish additional dimensions in health promotion roles alongside the three dimensions operationalized based on existing literature. Professional health promotion roles differ based on the following dimensions: (i) dealing with complexity, (ii) patient versus professional autonomy and (iii) involving client context in health promotion. First, in reframing, professionals clearly demarcate problems, while in customized health promotion the complexity of problems is embraced. Second, in reframing, professionals take the lead in deciding which problems are most appropriate to solve, while in customized health promotion professionals encourage clients to determine the direction of health promotion. Third, in customized health promotion involvement of the client context is more clearly observed.

### Professional identity

#### Professional identity 1: the pragmatic professional

Pragmatic professionals are professionals who see themselves as a fixers, who like to see impact of their work and who value setting boundaries around what they can and cannot do for a patient. Moreover, they value that they can do their job in ways they prefer, possibly by involving other professionals with different expertise. Additionally, these professionals respect clients’ solutions, but they also want clients to know about and to respect their professional suggestions.

**‘[I have] a focus on more pragmatic, hands-on work’** (Respondent A, GP)

*‘I think I am an all-round general practitioner, with a focus on more pragmatic, hands-on work. […] I am relatively more inclined to do things and less to have long conversations. […] Which means that I often do the more urgent care like injections and treatments and I think I’m also stronger in the musculoskeletal system. […] I like extreme medical cases. So, I can relish someone who’s living in a dirty house with rats and pus coming out of their ankle. There may be some general practitioners who think, ‘Yuck, do I have to go there?’. But there is often relatively a lot to do there, so there’s a relatively high impact of what you do.’*

This story is exemplary of pragmatic professionals, who *value* doing the type of work that they are trained for. They *believe* that they can reach their *goal* of making great impact.

Respondents with a pragmatic professional identity mainly use reframing health promotion roles and the data shows how this identity and this role interact. Respondents perform the reframing role as follows: first, by fixing something that they understand and that is manageable and preferably close to their professional strength. Second, by setting clear boundaries regarding what is and what isn’t their responsibility and professional scope. Aspects of the pragmatic professional identity, such as a drive to solve problems by using their professional expertise, are mobilized in taking up a reframing role. Respondents with a pragmatic professional identity sometimes use customized health promotion roles. This happens when their professional knowledge seems insufficient to understand the problem.

The pragmatic professional identity transcends the professions, and it occurs with professionals in social welfare and in general practice (see Supplementary Appendix E for more examples of health promotion roles from our data).

#### Professional identity 2: the holistic professional

A holistic professional is someone who is involved, listens and is motivated to take (extra) time and to extend the boundaries of their profession when this helps them to better help the client, especially those with complex problems. Therefore, this professional appreciates the collaborative aspect with the client on how to approach health promotion.

**‘much nicer [is] that someone is at your bedside who just cares about you’** (Respondent F, mental healthcare)

*What I find most important in my work is helping people who have a difficult life and that there is care and attention for them, also for people who may fall outside […] of what we think you should be as a citizen. […] I think that I would also want that when I would need care, that I would have a connection with [the caregiver]. I think that I would like to have a capable doctor, who knows what is technically the problem, but actually much nicer, that someone is at your bedside who just cares about you. I think that is worth a lot and if you find a balance in that, then I think you are the best care provider.’*

This story is exemplary of holistic professionals, who *value* being loyal caregivers with a present attitude towards their clients. Their *goal* is to be their authentic self, while being responsive in their interactions with clients.

Respondents with holistic professional identities perform the customized health promotion role as follows: (i) They emphasize that their solution does not necessarily have to align with the client’s solution. (ii) They prioritize being accessible to clients and work with tailored treatment rather then focused solely on diagnosis. (iii) They stress that boundaries can be complicated and that they sometimes go beyond what their role requires. For instance, respondent B (GP) enjoys that she now spends ‘*more time with clients that need it*’, she even holds longer conversations herselves, while other GPs usually delegate these to a practice assistant. Aspects of the holistic professional identity, namely taking time, being accessible, being flexible in how one approaches the task and being willing to shed boundaries, align with how these professionals play the customized health promotion role.

The holistic professional identity also transcends professions, as it occurs with professionals in social welfare, general practice and mental healthcare (see Supplementary Appendix E for more examples of professional identities from our data).

Two respondents with a holistic professional identity (respondents C and E, social workers) have scattered professional identities, which means that they identify as both holistic and pragmatic and that their professional identities are not very strong. This observation seems to results in the take-up of both professional roles (see Supplementary Appendix E for examples of professional identities and health promotion roles).

## DISCUSSION AND CONCLUSION

### Discussion

This research makes a threefold contribution to the health promotion literature. First, health promotion scholars do not acknowledge how professional identity shapes health promotion roles (Geense *et al*., 2013). What we learn from our research is that the health promotion roles of various professionals are more layered than descriptions of attitudes towards tasks, but that the actual roles performed relate to how one identifies as a professional, what one values and how one interprets one’s interaction styles. Insight into professional roles and professional identity brings the literature a step further in understanding why frontline professionals promote health of clients in certain ways. Moreover, in line with identity literature (Weick, 1995; Ashforth, 2000; Ashforth *et al.,* 2008), identity is a relational concept which is formed by promoting health with clients and other professionals. When and why various aspects are prioritized in health promotion in interaction with clients and other stakeholders are issues for future research.

Second, this research gives insight into the health promotion roles of various professionals instead of just those with medical backgrounds. This is relevant considering various frontline professionals collaborate to solve complex problems. Our findings indicate that both health promotion roles and professional identities transcend professional groups. This means that professional background does not determine one’s professional identity and health promotion role. This finding suggests that when collaborating across professions, professionals should potentially not be hindered by their different backgrounds. However, how these professionals are able to work together across professions goes beyond the scope of this research. Notwithstanding these findings, professional identity aligns with health promotion roles and some aspects of the professional background seem to play out in health promotion. First, consistent take-up of health promotion roles of professionals in social welfare seems to be challenged by their scattered professional identities emerging from factors such as NPM and gendered presumptions (Healy, 2009). Second, professional identities of professionals in mental healthcare align with their historical nature to focus on an authentic connection with the client (Schubert *et al*., 2021). Third, GPs, who are highly professionalized and are generally focused on the whole person, seem to have strong professional identities (McDonald *et al*., 2008), which plays out in clear adoption of health promotion roles. Additionally, in line with the literature on holistic GPs, GPs are holistic by nature. Holistic GPs focus on conventional methods when this is deemed more practical, for instance, when they interpret that the situation, such as a life-threatening situation, requires them to do so (Raaphorst and Houtman, 2016).

Third, based on our findings, we argue that health promotion roles can be conceptualized in more abstract ways than suggested in earlier research. Besides descriptions of tasks, health promotion roles in earlier literature differ based on the dimensions: type of involvement, perceived abilities and perceived importance of health promotion. The types of health promotion roles in our study, however, differ based on how professionals manage complexity, client autonomy and how they involve the client context in health promotion. These empirical findings resonate with broader developments in healthcare from reactive to proactive care, from cure to care and from disease-centered to patient-centered care as described for GPs (De Valck *et al*., 2001; Waldman and Terzic, 2019).

Health promotion roles pose several risks and advantages for clients and professionals. First, reframing health promotion is risky when working with a problem that a client does not see as a problem. This could mean that clients do not feel taken seriously, which could negatively impact the client–professional relationship and lead to unmet health needs of vulnerable clients (Salmon *et al.*, 2004). A potential advantage of reframing health promotion is that professionals who work in line with their professional expertise and skills are able to help clients. This could be valuable for clients and it could motivate professionals to solve clients’ problems. Second, a risk of power-sharing related to customized health promotion is that professionals put much trust in client autonomy, while not every client may be able to communicate their needs, and thus to take this responsibility. However, an advantage could be that clients feel heard, valued or even empowered and that they are helped in ways that align with their needs. Clients may experience empowerment to help themselves better. As such, our findings on the dimension of patient versus professional autonomy are in line with Larsen and Cecchini’s (2023) argument that professionals in healthcare need to be able to play dual roles, acting as traditional knowledge authority and also connecting on equal terms with clients’ views.

### Limitations

This research has limitations which need reflection. The goal of this research was to describe how professional identity shapes the health promotion roles of various professionals in social welfare, in mental and general healthcare. We aimed for a diverse sample with various professional backgrounds to unravel how professional identity, possibly triggered by professional background, shapes health promotion roles. The study’s methodological approach allows for theoretical rather than empirical generalization (Feldman and Orlikowski, 2011). To gain insight into possible patterns within and between the different professional groups, larger-scale research is recommended. Moreover, notwithstanding the strategy of snowball sampling does not guarantee representativeness (Johnson, 2014), this strategy was necessary to recruit respondents matching our theoretical selection: frontline professionals who work with clients with psychosocial problems. Thereafter, in the decision to use a specific health promotion role, other—contextual—factors may also play a role. Therefore, how health promotion roles are linked to personal identity, interprofessional collaboration and power-sharing with clients, should be further studied. Finally, future research should explore how clients experience health promotion.

### Conclusion

We have researched how professional identity shapes the health promotion roles of various professionals through an ethnographic study of frontline professionals promoting the health of clients with combined problems. This study contributes to the existing literature by describing professional identities and how they relate to practiced health promotion roles. Our expectation was that professionals with different professional backgrounds would identify differently and would therefore play different health promotion roles. Instead, we found that professionals’ health promotion practices are related to professional identities, which transcend professional backgrounds. Specifically, our findings indicate that frontline professionals in social welfare, general healthcare and mental healthcare promote health according to two roles: reframing health promotion and customized health promotion and that they identify as pragmatic and as holistic professionals. Even though the pragmatic professional identity is predominantly observed in professionals utilizing the reframing role and the holistic professional identity is prevalent among those engaged in customized health promotion, this relationship is not deterministic.

## Supplementary Material

daad195_suppl_Supplementary_Appendix
