# Supplementary material for: Health promotion roles shaped by professional identity: an ethnographic study in the Netherlands
Source: Health Promot Int. 2024 Jan 13;39(1):daad195. doi: 10.1093/heapro/daad195 (PMC10787352; doi:10.1093/heapro/daad195)
Supplement: daad195_suppl_Supplementary_Appendix [file daad195_suppl_supplementary_appendix.docx]

**Appendix A. Table with (main) respondents’ tenure**

| **Respondent number** | **Domain** | **Profession** | **Educational level** |
| --- | --- | --- | --- |
| A | Healthcare | General practitioner | University |
| B | Healthcare | General practitioner | University |
| C | Social welfare | Social worker or client supporter | Higher education |
| D | Mental healthcare | Mental health worker and social worker in mental healthcare | Higher education |
| E | Social welfare | Social worker with elderly people | Higher education |
| F | Mental healthcare | Social psychiatric nurse | Higher education |

**Appendix B. Observation guide with operationalization**

| **Health promotion role dimensions** | **Operationalisation** | **Example from fieldwork** |
| --- | --- | --- |
| *Type of involvement: reactive health promotion* | Code any text on a situation in which a frontline professional perceives a problem or symptoms to be clear and demarcated enough to respond to directly. During illness visits or conversations regarding specific symptoms, professionals educate or advise their patients about behavior, lifestyle or possible risks (McAvoy, Kaner et al. 1999). Reactive health promotion is firmly focused on disease risk-specific practices in favor of behavioral, disease-focused, lifestyle-oriented determinants of health. Such strategies fail to incorporate broader societal, economic, ecological, and political dimensions of health promotion (Runciman, Watson et al. 2006, Whitehead 2006, Casey 2007). | A client has clear symptoms of allergies and asthma and the GP reacts to this by prescribing medicine that should be used when specific symptoms appear. |
| *Type of involvement: proactive health promotion* | Code any text on a situation in which a frontline professional proactively performs an intervention without specific worrying symptoms in this direction expressed by the client. Professionals could even interfere in case of seeming high risk. Proactive health promotion could, for example, include proactively changing the problem in a way that the professional thinks is more relevant to the client (McAvoy, Kaner et al. 1999). Or by proactively trying to figure out what an underlying problem is. | 1. GP giving advice that does not directly fit with the problem the client came with. The GP thinks this is more relevant to the client or that this is what the client could mean.  2. GP interfering in someone’s life by giving unsolicited advice about, for example, the use of birth control for someone at high risk of unwanted pregnancy. |
| *Perceived ability health promotion role: able* | Code text on frontline professionals who feel able, skilled and/or responsible to promote health of their clients. They feel they have the right knowledge and facilities to do so and they perceive it to be their task. | ‘It is my task to help with anything in the social environment. You can ask me anything, because it is my job to help you. Don’t hesitate.’ (social worker) |
| *Perceived ability health promotion: unable* | Code any text on frontline professionals who don’t feel able or skilled and/or not responsible for health promotion. | 1. ‘It is not my role, to write such nonsense statements, but no one else will do it.’ (GP)  2. ‘It is not our role to help you so comprehensively. It is your own responsibility to get to know how your phone works and how to use it for your finances.’ (Social worker) |
| *Perceived ability health promotion: collaborative* | Code any text on frontline professionals who do take on health promotion activities, but only when they can collaborate with other stakeholders. | ‘I think this client has real problems but I’m not sure how we can help them apart from listening to them. I refer them to the practice nurse so they can help find an experience expert to connect with.’ |
| *Perceived importance health promotion: emphasize* | Code any text on frontline professionals who emphasize the importance of health promotion activities. They are motivated and willing to promote health. | ‘One thing that really motivates me in my work is to help people get healthy, to care.’ (GP) |
| *Perceived importance health promotion: skeptical* | Code any text on frontline professionals who are skeptical about health promotion and its results. They expect that it won’t really help and are thus neither motivated nor willing. | ‘If I help them with this task, then they will never learn to do it independently and they will come back over and over again.’ (Social worker) |
| Professional identity aspects | Operationalization | Examples from fieldwork |
| *An individual’s self-definition* | Code any text on an individual’s self-definition as a member of a profession (Adams, Hean et al. 2006, Chreim, Williams et al. 2007). | ‘I identify as a real caregiver in heart and soul.’ (Mental healthworker). |
| *Professial uniqueness* | Code any text on what makes the professional unique on their own and how they become meaningful relative to others through clear goals, norms, beliefs, values, interaction styles and member interdependencies that are associated with a role in work situations (Ashforth 2000). | ‘Its is my goal that when people leave here they they feel lighter. It makes no sense to judge, therefore I go into what people find important.’ (Mental healthworker). |
| *Cultural expectations* | Code any text on the cultural expectations about how to behave in a social position (Burke and Stets 2009). | ‘I like to indicate boundaries around my professional expertise, towards clients and towards other professionals. I would rather do what I am good at.’ (Mental healthworker). |

**Appendix C.**

**Table with hours of observation per respondentgroup**

| **Respondent group** | **Hours of observation** | **Days of observation** | **Formal semi-structured interviews** |
| --- | --- | --- | --- |
| Mental healthcare | 65 hours | 14 days | 1 |
| General healthcare | 45 hours | 11 days | 1 |
| Social welfare | 40 hours | 9 days | 1 |

**Appendix D.**

**Conversation/interview guide**

| *Briefing/appointments*  Discussing confidentiality, anonymity and introduction to interview  *Professional roles*  What is your professional background?  What is your work experience like?  What are your core professional roles? |
| --- |
| *Health promotion*  What are health promotion roles (or not) according to you?  What does heatlh promotion mean to you?  Do you believe health promotion is a core task as a professional? Why/ why not? What is more important? |
| *Professional identity*  How would you describe yourself as a professional?  How do you value these roles and tasks (or not)?  What meaning do you give to these roles and tasks?  What do you find most important in your work with clients with combined psychosocial problems?  What are you good at, what do you contribute to your job?  Do you think you are competent/ the right stakeholder to work on health promotion? Why/ why not? |
| *Motivations*  Why do you play these roles (or not)?  Why did you handle this case in this way? (follow-up question during observation) |
| *Other follow-up questions*  What did you do here?  Why did you do this?  How do you see your role in this situation? |
| *End of interview*  Do you want to add something?  How did you experience this interview? |

**Appendix E.**

**Persona**

| Respondent A, professional in general healthcare |  |
| --- | --- |
| Observed health promotion roles | I use a *reframing health promotion* role |
| This is how I fulfill my role | I would rather just fix something that I understand and that is manageable and preferably medical, which aligns with my professional strengths. Otherwise, I can refer a patient so someone else who can offer help.  I fulfill my health promotion role by setting boundaries regarding what is and isn’t my scope of responsibility and I consider it important to focus on the aspects that align with my professional strengths. |
| Example of how I fulfill my professional role in health promotion | ‘*If I disagree with [a patient about wanting antibiotics], I sometimes still prescribe antibiotics, but at least I have had my say. I provide advice based on what I consider important, but I am accommodating. I don’t engage in constant debates, as work should also remain enjoyable*.’ |
| My professional identity is | The *pragmatic professional*  I am a pragmatic general practitioner, a fixer, I like extreme medical cases, I value setting boundaries around what I can and cannot do for a patient and I value that we can manage our general practice as a business. |
| Example of my professional identity | The pragmatic professional  *‘Yes, I think I am an all-round general practitioner, with a focus on more pragmatic, hands-on work. That means that I am relatively more inclined to do things and less to have long conversations. […] Maintenance of psychiatry, I have somewhat less affinity with that. […] Which means that I often do the more urgent care like injections and treatments and I think I’m also stronger in the musculoskeletal system. […] I like extreme medical cases. So, I can relish someone who’s living in a dirty house with rats and pus coming out of their ankle. Yes, so I feel like, nice. There may be some general practitioners who thinks, ‘Yuck, do I have to go there?’. But there is often relatively a lot to do there, so there’s a relatively high impact of what you do. It’s a combination of wonder, the bizarreness of the syndrome, or the extreme aspects, I find that interesting and intriguing. How someone ended up in such a situation and what the background is. Yes, so that, and the same goes for when people are really seriously ill, I often find that fascinating. It just becomes more medically interesting, I think. So someone who says, ‘No, I’m not feeling quite right in my well-being,” I find less interesting than someone who is really in the midst of a big psychosis, constructing entire theories about how they are going to improve the world… I find a genuine first psychosis to be a very, very interesting medical picture.’’* |

| Respondent D, professional in mental healthcare |  |
| --- | --- |
| Observed health promotion roles | I use a *customized health promotion* role |
| This is how I fulfill my role | I fulfill my health promotion role by prioritizing being accessible to patients through platforms like WhatsApp. I furthermore do so by working with tailored treatment rather than focused solely on one diagnosis. I help patients in the way that works for them. The goal is that people leave here feeling like a heavy burden has been lifted off of them. |
| My professional identity is | The *responsive professional 🡪 holistic*  I am an accessible, responsive professional who values adapting to what the patient needs and who intends to foster a strong therapeutic relationship.  The *caring professional 🡪 holistic*  I am someone who truly wants to assist people that are really in need. |
| Example that shows my professional identity | The holistic professional  The respondent is undergoing training as a Cognitive Behavioral Therapist, and she finds it very informative. She expresses that this should be mandatory, stating, ‘*My conversational techniques are now very different. Instead of just skirting around issues, I now have more knowledge to really address and assist. It’s transformative. What I find important is to be there for people, especially those with multiple diagnoses. While there is a lot of stigma about our patient group, I think we can really help them here. In finding a solution I think it is really valuable that we can work on finding a fitting treatment here instead on just focusing on one diagnosis like depression. […] I will try to go with what works for the patient, it doesn’t have to be my solution. […] Thereby, I try to protect the patient, both from themselves and from other professionals.*’ |

| Respondent F, professional in mental healthcare |  |
| --- | --- |
| Observed health promotion role | I use a *customized health promotion* role |
| This is how I fulfill my role | I fulfill my health promotion role by letting the patient know that I am there as a consistent support for them instead of wanting to fix things. The essence of my work lies in ensuring that everyone has someone who cares for them, looks out for them, and shows concern.  And by building a connection without immediately wanting to judge or solve things. The reason being that ‘*every individual’s journey in receiving care is unique. I might tidy up here, but that would not be helpful for her. We are working on recovery in different ways*’. |
| My professional identity is | The *caring* professional 🡪 holistic  I am a natural- born caregiver.  The *present* professional  I value being present for my patients and their needs. Thereby, I bring genuineness, authenticity and loyalty into my interactions. |
| Example that shows my professional identity | The holistic professional  ‘*The presence approach, yes […]. That’s actually kind of the basis of what I do, within the pressure of business and management. We have a big caseload with complex cases. But being there for people and keeping a part of my agenda free to map out the worrying cases, to have some sort of free space to ring the doorbell three times. It is not possible teamwise, but Ideally I reserve a few hours a day for this. This way I can really invest in a relationship without immediately providing assistance, but based on being there for people and listening to what someone needs and just radically being there for them without judgment. Presence theory is thus the basis of providing good care, by getting to know someone well first. Based on that understanding and that very strong relationship, you can get very far.’*  ‘*I’m not different as a human than as a professional, only the profession sits over it like a layer. And of course a few things that I am or I am not in my private life, you obviously don’t take with you in your profession. But that authenticity and that I make contact with people and this authenticity they feel that. And the loyalty and being there for someone. […] So I’m always myself, I’m the nurse and the care provider at work, while I’m at home I’m also normal.* *Well, in my private life I also take care of other people and then you can say that in any case I’m a social person, I don’t know how to say it, but it is not that different from how I am in real life, but I think that is quite necessary because the [clients] then feel that it is serious, and that you are not coming to pretend, or play a game or something. […]* |

| Respondent B, professional in general healthcare |  |
| --- | --- |
| Observed health promotion roles | I use a *customized health promotion* role |
| This is how I fulfill my role | I fulfill my *customized* health promotion role by having informal conversations, *building a relationship*, and trying to *understand the patient’s environment*. For instance, ‘I ask many questions to the patient to gain a better understanding of the situation and to clarify any uncertainties that may be related to the problem. This way, I aim to uncover the true nature of the issue at hand and the right approach by taking the patient and their concerns seriously. I provide them with the opportunity to (re)gain control over the care process.’  I fulfill my customized health promotion role by doing more when this is necessary. For example, by ‘engaging in conversations while someone waits for specialized psychological help.’ |
| Example of customized health promotion role in data | The client’s mother explains that the doctor’s file says it is jaundice, but that this ‘*is not the case […]. [The child] suffers from a painful stomach and ribs, and has thin stools every day*’. The professional asks if there are things that the child cannot do because of her complaints. The child suffers during gymnastic class, where she cannot participate without pain. The respondent asks follow-up questions to figure out when and how the child suffers and how the mother observes this. Then the respondent explains that it is difficult to figure out what is going on based on what they know. There are many complaints but there is no clear pattern. They agree to do serveral more tests during the next appointment. Respondent to client and mother: ‘*It’s always a matter of weighing up together what is more annoying, all these tests or the complaints*.’  This story is exemplary of customized health promotion in which professionals find it *important* to work closely with clients and their enviroment by asking questions to figure out what is important to the client regarding their physical complaints, but also in other life areas. |
| My professional identity is | The *holistic* professional  I am an involved professional who listens, and who is motivated to and interested in solving complex issues. I am willing to extend the boundaries of my profession when this helps me to better help the patient. |
| Example that shows my professional identity | The holistic professional  ‘*That I let people take control themselves. Yes, I think that also reflects a bit on how I approach life. Of course, that says something about me as well. […] That’s the interesting aspect of our general practitioner profession- where does the boundary lie between what is within the realm of a general practitioner, and where does the line between societal responsibility and your role interpretation lie? I think that’s the beauty of our profession; every general practitioner has to determine that for themselves. There’s no right or wrong, but it varies for everyone.* *I can easily imagine that there are colleagues who say ‘You come here with back pain, so I only treat the request present to me’. That is also very legitimate. For me, it’s slightly different if it turns out that the person keeps coming back with that back pain and apparently isn’t helped with the answer I give to the initial request. Then I want to explore further. […] [What I find enjoyable about my job here is that] it’s about the whole concept of humanity, I think. […] It is clear that health, for me, is not only physical. It’s also about how people function in other life domains. In that sense, the holistic nature of the general practitioner profession is what I chose many years ago. […] That is the basis of the general practitioner profession, I believe. The strength of our profession is that we have the opportunity to get a much broader view of those life domains because each domain influences health*.’ |

| Respondent E, professional in social welfare |  |
| --- | --- |
| Observed health promotion roles | I use *customized and* *reframing health promotion* roles |
| This is how I fulfill my roles | I fulfill the *customized* health promotion role by making sure that ‘*my solution doesn’t have to be theirs*.’ ‘*However, boundaries can be complicated, and sometimes I go beyond what is strictly required for a client. When a client comes here in distress, I will not turn them away. At the same time, I find it important that clients take responsibility and initiative*.’  I fulfill the *reframing* health promotion role by helping with everything related to the social aspect. For instance, ‘*this woman came here with pain issues, but I think she is actually afraid and lonely and she should start volunteering again. […] We are from prevention, so these are things that we should take notice of.’’* |
| Example of reframing health promotion role from data | The professional and I greet the woman who sits in her mobility scooter when we arrive outside her building. She had forgotten that the social worker would visit today, but she says that she is happy that we are here. We walk and talk together for more than an hour. The client says that she: ‘*would like to keep walking with her until […] [she] can walk independently again*.’ Later, the professional tells me that: ‘*She will probably never walk independently again and I cannot help with the instability in her legs. […] I think this is actually a loneliness issue. When I have not visited her for a week I can really see that she is lonely, depressed and sad and she really feels better after I came by. Walking with her is a way for me to talk with her and to monitor her social isolation’.*  In this example, respondent E reframes a physical problem of not being able to walk independently due to a sore leg into a problem of social isolation. The respondent understands that the client experiences insecurity when walking due to pain in her legs. As a social worker, the respondent is not able to help with the legs, but she can help with a related social problem: loneliness. |
| My professional identity is | The *proactive* professional 🡪 pragmatic  I am a doer, which means that I am driven to get things done. I go the extra mile for a client and I push boundaries to make things happen for a client.  The *respecting* professional 🡪 pragmatic  I value respecting the clients’ solution, but they should also respect my professional suggestions.  The *flexible* professional 🡪 holistic  What I appreciate about this job is the flexibility to shape how I approach each task and focused on which domain […]. |
| Example that shows my professional identity | The pragmatic professional  ‘*I work on everything related to the social.* *Sometimes I think, if you put in a little extra effort, you can get people over a hurdle, to something, and then you can actually help people*. […] *Well, people don’t need to get down on their knees or bring flowers, like, ‘oh, thanks.’ Just seeing how people progress or when they say ‘you’ve really helped me overcome my fear of public spaces, you know, by taking me out with someone,’ then I find it okay. But don’t take me for a ride [when I have put a lot of work in your care]*.’  The holistic professional  *‘In that sense, I can really empathize with how it is for people, that you can really feel lost. Well, and that does create a bond. […] If I know it helps, I mention [that I have been ill too and how I dealt with that]. […] Well, I think I’m a good listener. I find it important to pay attention to the client. I try not to force my solution down their throat, so I listen to their problem and my solution doesn’t have to be the client’s solution.’*  *‘And this was not my task, but I think this is also social work. That’s what I appreciate about this job, that I can shape my role the way I want to.’* |

| Respondent C, professional in social welfare |  |
| --- | --- |
| Observed health promotion roles | I use *customized and* *reframing health promotion* roles |
| This is how I fulfill my roles | I fulfill the *customized* health promotion role by having longer conversations during one on one appointments with clients who are able to express their problems. ‘*Then, I enjoy assisting with psychosocial issues*.’  I fulfill the *reframing* health promotion role by assisting people in addressing their material concerns to the extent of our capabilities. ‘*During the open office hours, we only do short social questions. Then, professional boundaries are central to me, otherwise, I end up dealing with minor tasks that are not within the scope of my education*.’  However, often, ‘*I cannot really fix their problems, but all I can do is listen*.’ |
| My professional identity is as follows | The eager professional 🡪 pragmatic  I am eager to help when I can truly make a meaningful impact for a client who takes ownership of their own wellbeing.  The eager professional 🡪 holistic  What motivates me is when I can help people by having longer conversations about their problems that go beyond just material stuff. What I really like is to listen, when I know I can really mean something by empowering them. |
| Example that shows my professional identity | The pragmatic professional  ‘*I can’t help someone who doesn’t take ownership of solving their own problems. Otherwise, I end up doing small tasks that I haven’t studied for. It’s important for me to set boundaries on what is and isn’t my responsibility. When a client pressures me, I won’t work harder. I want to help, but only if I feel like I can truly make a difference. […] This profession is not what it has been, I don’t feel taken seriously anymore. I feel like I can’t do the work that I want to do*.’  The holistic professional  ‘*Addressing relationship issues is a significant aspect of my work because it can have a profound impact on someone’s life, relationship problems*.’ |
